# Surgical management of meningioma in a resource-limited setting: a case report

**DOI:** 10.1097/RC9.0000000000001050

**Published:** 2026-09-29

**Authors:** Rugendabanga K. Edouard, Denis M. Ndeni

**Affiliations:** a Department of Surgery, General Surgery, Muhimbili National Hospital, Dar-Es-Salaam, Tanzania; b Department of Surgery, Hôpital Général de Référence de Panzi (HGRP), Université Evangelique en Afrique (UEA), Bukavu, Democratic Republic of the Congo

**Keywords:** case report, delayed diagnosis, meningioma, resource-limited settings, Simpson Grade I, WHO grade

## Abstract

**Background:**

Meningiomas represent the most prevalent primary intracranial neoplasms, accounting for approximately 30% of all intracranial tumors, with 90% classified as benign (World Health Organization [WHO] Grade I). Surgical resection remains the primary therapeutic modality. However, in low-income and conflict-affected regions, timely diagnosis and surgical intervention are frequently constrained by limited access to neuroimaging and specialized neurosurgical care.

**Case presentation:**

We present the case of a 37-year-old male who developed progressive neurological deterioration over 6 months. Clinical features included left-sided hemiparesis, aphasia, seizures, right-eye blindness, and sphincter incontinence. Cranial computed tomography (CT) revealed a right fronto-parietal extra-axial mass with substantial peritumoral edema, subfalcine herniation, and probable transtentorial involvement. The patient underwent gross total resection of a 5 cm encapsulated tumor via craniotomy. Histopathological examination confirmed a WHO Grade I meningioma composed of epithelioid cells arranged in clusters, interspersed with fibroblast-like cells. Postoperative recovery was favorable, with partial neurological improvement and persistent visual impairment.

**Discussion:**

This case highlights the challenges associated with the diagnosis and management of intracranial meningiomas in resource-limited and conflict-affected settings, where delayed presentation and restricted access to neuroimaging frequently result in advanced neurological deterioration at the time of diagnosis. Despite the absence of several standard neurosurgical resources, including preoperative magnetic resonance imaging and neuronavigation systems, successful surgical management was achieved through careful CT-based anatomical planning and meticulous microsurgical technique. Histopathological examination confirmed a WHO Grade I meningioma with a low MIB-1 (Ki-67) labeling index (<3%), supporting a favorable long-term prognosis following Simpson Grade I gross-total resection.

**Conclusion:**

This study demonstrates how care can be provided in a constrained environment using a preoperative CT scan and a Simpson Grade I excision. There was an improvement in symptoms, but visual loss could not be recovered.

## Introduction

Meningiomas, which arise from the arachnoid cap cells of the meninges, represent the most frequently diagnosed primary intracranial neoplasms, accounting for approximately 30% of all central nervous system tumors^[^1^]^. The vast majority are classified as World Health Organization (WHO) Grade I, characterized by a benign biology and slow growth, making them potentially curable with complete surgical resection^[^2^]^. The extent of this resection, classically defined by the Simpson Grading Scale, remains the single most critical prognostic factor for preventing recurrence^[^3^]^.

HIGHLIGHTS

- Strengthening local neurosurgical services.
- It aims to illuminate the unique challenges and strategic adaptations necessary to deliver complex neurosurgical care in a resource-limited context.
- Immediate positive outcomes can be observed, especially in World Health Organization Grade I cases.

In high-income countries, the management of meningiomas is a streamlined process underpinned by sophisticated diagnostic tools such as magnetic resonance imaging (MRI), advanced microsurgical techniques, and multidisciplinary neuro-oncological care. This infrastructure facilitates early detection and intervention, often before the onset of severe neurological deficits. Where these facilities are not available, a CT scan can be used to plan surgery, and no postoperative imaging may be necessary when dealing with WHO Grade 1 tumors. Surgical resection achieving a Simpson Grade I excision (Table 1), defined as complete macroscopic removal of the tumor together with resection of its dural attachment and any involved adjacent tissue, thereby provides the lowest expected risk of recurrence (Table 2). Regarding the recurrence rate, a long-term postoperative plan can be made, but it is not mandatory when Simpson Grade I excision has been achieved.

**Table 1:** Simpson Grading System for meningioma resection.

Simpson Grade	Description of resection	Key surgical actions	10-year recurrence rate
Grade I	Macroscopically complete removal; excision of dural attachment and any abnormal bone.	The tumor, its dural base, and any infiltrated bone (e.g., hyperostotic skull) are removed. This often requires dural plasty.	~9%
Grade II	Macroscopically complete removal; coagulation of dural attachment.	The tumor is fully removed, and its dural base is thoroughly coagulated with bipolar cautery to destroy residual cells.	~19%
Grade III	Macroscopically complete removal; without resection or coagulation of dural attachment.	The tumor itself is fully removed, but the dural base of attachment is left untreated (no resection, no coagulation).	~29%
Grade IV	Subtotal removal; leaving tumor *in situ*.	Only a portion of the tumor is removed, leaving macroscopic (visible) tumor behind, often to protect critical neurovascular structures.	~39%
Grade V	Simple decompression (biopsy only).	Only a biopsy is performed for diagnosis, with or without a decompressive procedure to relieve pressure.	N/A (recurrence is certain if not treated)

**Table 2:** WHO Classification of meningiomas.

WHO grade	Category/subtype	Key features
Grade I (benign)	- Meningothelial	- Most common (≈80%)
	- Fibrous (fibroblastic)	- Slow-growing
	- Transitional (mixed)	- Low recurrence after complete resection
	- Psammomatous	
	- Angiomatous	
	- Microcystic	
	- Secretory	
	- Lymphoplasmacyte-rich	
	- Metaplastic	
Grade II (atypical)	- Atypical meningioma	- Increased mitotic activity (≥4 mitoses/10 HPF)
	- Chordoid	- Brain invasion now automatically qualifies as Grade II
	- Clear cell	
		- Higher recurrence compared to Grade I
Grade III (malignant/anaplastic)	- Anaplastic meningioma	- High mitotic index (≥20 mitoses/10 HPF)
	- Papillary	- Aggressive behavior
	- Rhabdoid	- High recurrence and risk of metastasis

## Case presentation

A 37-year-old right-handed male with a 15-year history of ingesting highly alkaline beverages presented with a 6-month history of intermittent fever and progressive neurological symptoms. These included left hemiparesis (predominantly affecting the lower limb), global aphasia, monocular blindness in the right eye, generalized tonic-clonic seizures, and urinary and fecal incontinence.

The patient was initially evaluated at a neuropsychiatric center and subsequently admitted to an internal medicine service, where a positive rapid diagnostic test for *Plasmodium falciparum* and the presence of cerebral malaria guided antimalarial therapy for complicated malaria (Table 3).

**Table 3:** Timeline table.

Timeline	Clinical events and interventions
6 months before admission	Progressive onset of neurological symptoms including left-sided hemiparesis, predominantly affecting the lower limb, recurrent generalized tonic-clonic seizures, progressive aphasia, right eye visual loss, and urinary/fecal incontinence.
3 months before admission	Patient evaluated at a neuropsychiatric center and later admitted to the internal medicine department. A rapid diagnostic test was positive for *Plasmodium falciparum*, and treatment for complicated cerebral malaria was initiated.
One day before admission	Non-contrast cranial CT scan demonstrated a right fronto-parietal extra-axial dural-based mass measuring approximately 5 cm, associated with extensive vasogenic edema, subfalcine herniation, and early transtentorial shift.
On admission day	Severe functional impairment with estimated Karnofsky Performance Status (KPS) score of 40. Motor examination revealed left-sided weakness with Medical Research Council (MRC) grades of 2/5 in the lower limb and 3/5 in the upper limb.
2 days after admission	The patient underwent emergency frontotemporal craniotomy with gross total resection of the tumor under general anesthesia.
Day 1 post-surgery	Physiotherapy rehabilitation.
3 months post-suregry	Patient completed 20 physiotherapy sessions with progressive neurological recovery.

A non-contrast cranial CT scan revealed a right fronto-parietal extra-axial, dural-based mass measuring approximately 5 cm in diameter, with significant surrounding vasogenic edema, subfalcine herniation, and early transtentorial shift (Fig. 1).

**Figure 1.**
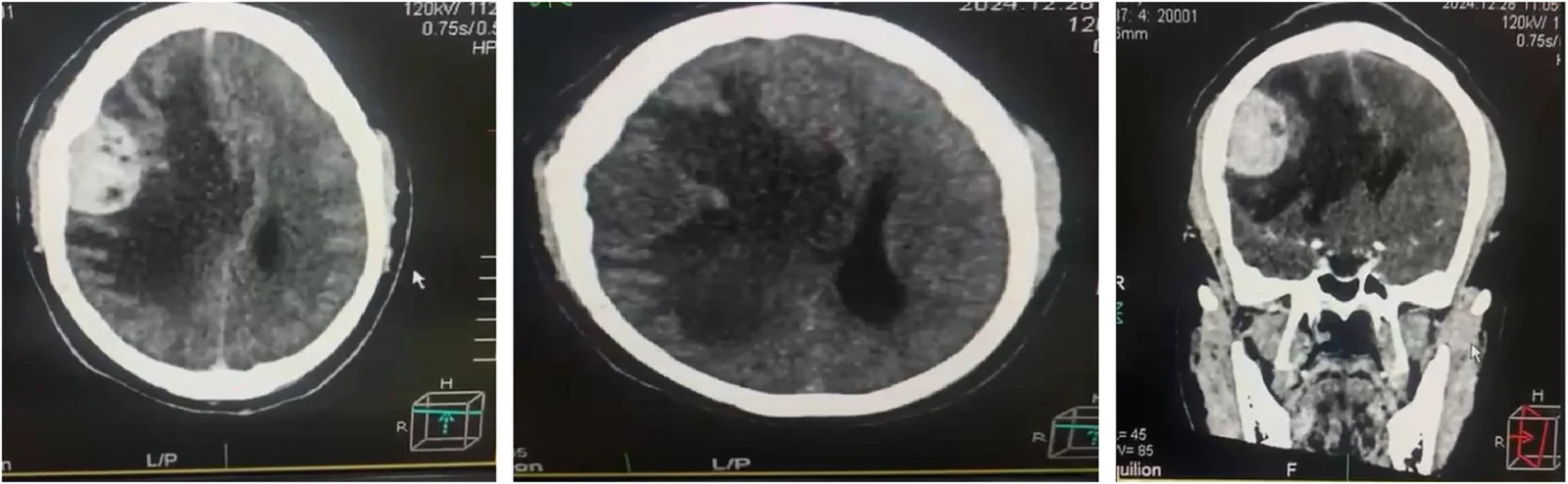
CT scan of the brain.

The patient underwent an emergency craniotomy under general anesthesia. Intraoperatively, a well-circumscribed, firm, and vascular mass was resected en bloc.

## Operative details

Following induction of general anesthesia, the patient was positioned supine. The head was fixed in a Mayfield skull clamp, rotated approximately 30° to the left, and elevated to promote venous drainage.

A standard question-mark-shaped pterional incision was marked and infiltrated with a solution of 1% lidocaine with epinephrine. After standard sterile preparation and draping, the skin incision was made. The superficial temporal artery was identified and preserved. The temporalis muscle was incised and reflected inferiorly in an anatomic layer, exposing the frontotemporal bone.

A frontotemporal craniotomy was performed with four burr holes. The bone flap was elevated and removed. The dura was noted to be under tension and non-pulsatile. Under microscopic visualization, the dura was opened in a C-shaped fashion and reflected anteriorly over the sphenoid wing. Initial inspection of the cortex revealed slight effacement of gyral patterns in the mid-frontal region. A limited corticectomy of approximately 2 cm was performed posterior to the motor strip.

Upon entering the white matter, a firm, rubbery, well-encapsulated mass was encountered. The tumor was grayish-tan and distinct from the surrounding edematous brain parenchyma. It was notably avascular. A clear surgical plane was developed circumferentially around the lesion. Using meticulous microsurgical technique, including bipolar cautery and sharp dissection, the tumor was carefully dissected free from its attachments. The anterior portion of the mass was adherent to the overlying dura, which was resected en bloc with the tumor. The lesion was delivered in its entirety, achieving a gross total resection. The surgical bed was then meticulously inspected, and hemostasis was secured with bipolar coagulation and the application of a topical hemostatic agent (Surgicel).

The dural defect was closed in a watertight fashion with a running 4-0 Prolene suture. The dura was then tented centrally to the bone edges to minimize the risk of epidural collection. The bone flap was re-affixed using a titanium cranioplasty plate system. The temporalis muscle and fascial layers were re-approximated with interrupted absorbable sutures (2-0 Vicryl). A subgaleal closed-suction drain was placed, and the scalp was closed in two layers. A sterile dressing was applied.

The patient was subsequently extubated and transferred to the neurosurgical intensive care unit in stable neurological condition, moving all extremities spontaneously. Postoperative management consisted of analgesia with intravenous paracetamol 1 g every 8 hours and Tramadol 100 mg twice daily; Anti-edema Therapy: Intravenous Methylprednisolone (Solumedrol) 120 mg daily; and seizure prophylaxis: Levetiracetam 1000 mg twice daily.

Histopathological analysis of the resected specimen confirmed a WHO Grade I meningioma. The tumor was composed of densely packed epithelioid cells arranged in whorls and clusters; immunohistochemical analysis demonstrated a low proliferative index, with an MIB-1 (Ki-67) labeling index estimated at less than 3%, supporting the diagnosis of a WHO Grade I meningioma with low proliferative potential. No atypical mitotic activity, necrosis, or evidence of brain invasion was identified.

Postoperatively, the patient underwent 20 physiotherapy sessions. Motor strength progressively improved following rehabilitation, from a preoperative Medical Research Council grade of 2/5 in the left lower limb and 3/5 in the left upper limb to 4 +/5 strength in both extremities at follow-up. However, visual loss in the right eye persisted.

The patient was referred to ophthalmology, and they reported that visual acuity was reduced to light perception only bilaterally, associated with semi-mydriasis of the right eye. Intraocular pressure was measured at 12.4 mmHg bilaterally. Fundoscopic examination revealed no significant abnormalities. Given these findings, retrobulbar optic neuropathy is strongly suspected. Brain and orbital MRI was recommended to further characterize the lesions and assess for possible involvement of the optic pathways. Unfortunately, the patient could not afford it.

The patient was very satisfied that we improved his quality of life. He was concerned about long-term follow-up, as he might not be able to comply.

## Discussion

This case illustrates the successful surgical management of a large intracranial meningioma in a setting constrained by limited diagnostic and therapeutic resources. The patient presented with advanced neurological symptoms, a common occurrence in low-resource settings due to delayed presentation and lack of access to neuroimaging, as reported by Young *et al*^[^4^]^.

While MRI remains the gold standard for preoperative evaluation of intracranial masses, CT imaging, when available, can provide critical information to guide surgical intervention, as shown by Saloner *et al*^[^5^]^. In this case, CT imaging was instrumental in identifying mass effect, herniation, and edema, justifying urgent surgical decompression.

Histologically, WHO Grade I meningiomas are associated with a favorable prognosis following gross total resection. The MIB-1 (Ki-67) labeling index is increasingly recognized as an important prognostic biomarker in meningioma management. Although WHO Grade I meningiomas generally demonstrate favorable long-term outcomes following gross total resection, an elevated Ki-67 index (>5%) has been associated with increased recurrence rates and shorter progression-free survival. In the present case, the low Ki-67 index (<3%) supports a favorable long-term prognosis, although continued radiological surveillance remains necessary^[^6^]^.

Molecular profiling has also emerged as a promising adjunct in modern meningioma management. Mutations involving NF2, SMO, AKT1, TRAF7, and KLF4 have been associated with distinct biological behaviors and anatomical subtypes of meningiomas. Although molecular testing was unavailable in our setting, the incorporation of molecular diagnostics into future practice may help guide prognostication and individualized therapeutic strategies, particularly in atypical or high-grade tumors^[^6^]^.

The absence of intraoperative complications and the patient’s substantial neurological recovery attest to the potential for effective neurosurgical care^[^7^]^. Without any imaging or laboratory investigations, the team relied on the patient’s modified Rankin Scale score. The patient’s modified Rankin Scale score improved from 5 preoperatively to 3 following surgery and rehabilitation^[^8^]^.

## Conclusion

This case unequivocally demonstrates that life-saving and transformative neurosurgical care is achievable even in the most austere and low-income regions. The case involved the successful gross total resection of a large meningioma.

## Strengths and limitations

### Strengths

This report highlights the successful management of a large intracranial meningioma in a severely resource-limited and conflict-affected setting, demonstrating that complex neurosurgical procedures can be safely performed despite major infrastructural constraints. The case provides a detailed description of the clinical presentation, radiological findings, operative management, histopathological features, and postoperative rehabilitation course. In addition, we achieved a Simpson Grade I resection with favorable neurological recovery.

### Limitations

Preoperative MRI, neuronavigation, intraoperative neurophysiological monitoring, and molecular profiling were unavailable because of resource constraints, limiting comprehensive preoperative characterization and advanced surgical planning. Long-term postoperative radiological follow-up could not be fully guaranteed, potentially limiting accurate assessment of recurrence risk. Furthermore, the absence of advanced ophthalmologic imaging limited detailed evaluation of the persistent visual impairment and suspected retrobulbar optic neuropathy. As a single case report, the findings may not be generalizable to all neurosurgical settings within low- and middle-income countries.

## Recommendations

1. Improving access to neuroimaging: the expansion of affordable CT and MRI services in underserved regions is essential for earlier diagnosis and timely referral of patients with intracranial tumors.
2. Enhance pathology and molecular diagnostics: Establishing access to immunohistochemistry and molecular profiling may improve tumor classification, prognostication, and individualized treatment planning.
3. Develop multidisciplinary rehabilitation services: Early physiotherapy, occupational therapy, speech therapy, and neuro-ophthalmologic care should be integrated into postoperative management to optimize functional recovery.

Artificial intelligence was used to convert the work from French into English and to rephrase some sentences in a more formal way. ChatGPT was used for this purpose.

The patient was operated on by a neurosurgery specialist and a second-year general surgery resident.

### SCARE guideline

The work has been reported in line with the SCARE criteria. The work was conducted following the SCARE guidelines.

## Data Availability

The datasets of the present study are available from the corresponding author upon request.
